# Supplementary material for: Comprehensive benchmarking of large language models for RNA secondary structure prediction
Source: Brief Bioinform. 2025 Apr 10;26(2):bbaf137. doi: 10.1093/bib/bbaf137 (PMC11982019; doi:10.1093/bib/bbaf137)
Supplement: rnallm_supp_bbaf137 [file rnallm_supp_bbaf137.pdf]

Comprehensive benchmarking of large language models for RNA secondary structure prediction

L.I. Zablocki, L. A. Bugnon, M. Gerard, L. Di Persia, G. Stegmayer, D. H. Milone

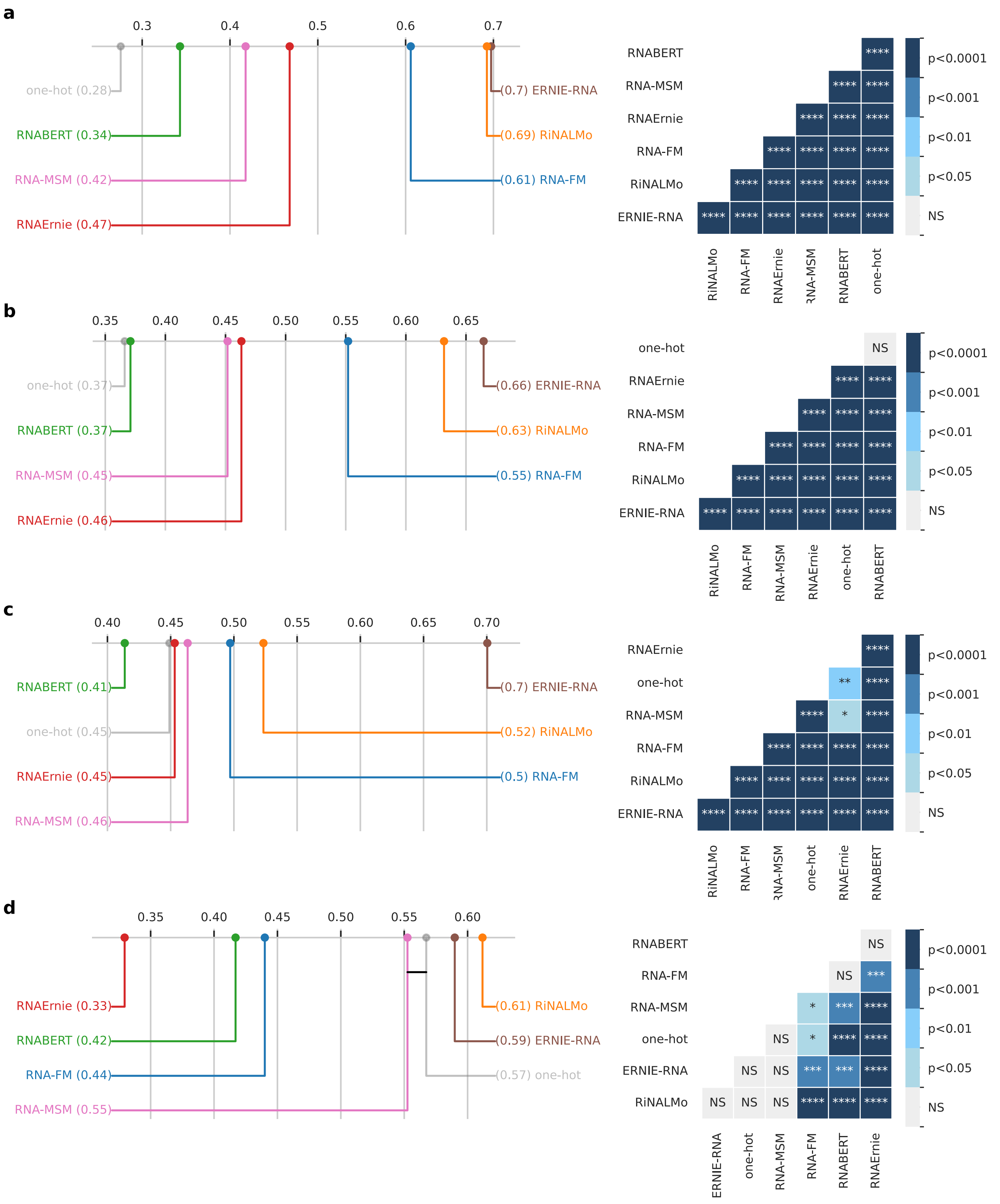

Figure S1. Statistical analysis for RNA-LLM on the RNA secondary structure prediction task for different benchmark datasets of increasing complexity. Left: Friedman test and critical difference with post-hoc Nemenyi test with Bonferroni correction (1). Right: Friedman test with Wilcoxon signed-rank test for paired samples with Bonferroni correction as post-hoc test (2). **a**, ArchiveII 5-fold random cross-validation. **b**, bpRNA train-test partitions with controlled homology. **c**, bpRNA-new dataset, for RNA families not seen during training. **d**, PDB-RNA dataset, with RNA sequences extracted from PDB.

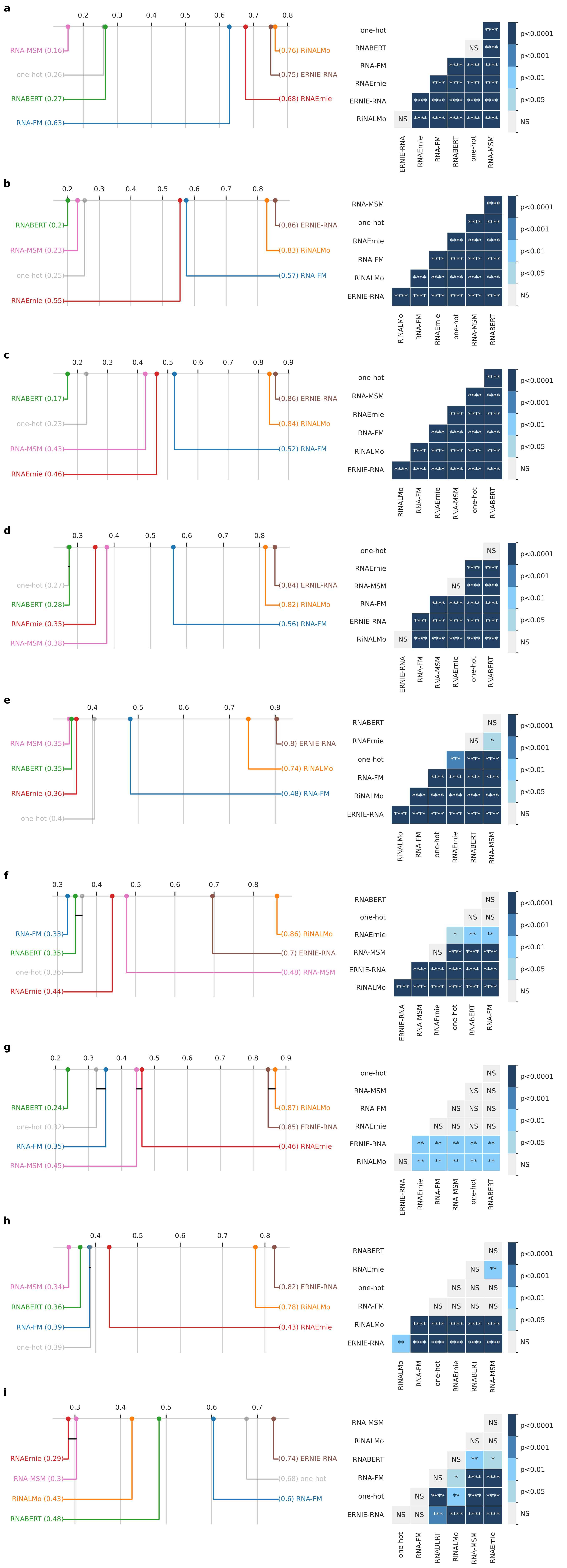

Figure S2. Statistical analysis for the inter-family structure prediction based on RNA LLM. Left: Friedman test and critical difference with post-hoc Nemenyi test with Bonferroni correction (1). Right: Friedman test with Wilcoxon signed-rank test for paired samples with Bonferroni correction as post-hoc test (2). **a**, tRNA family. **b**, 5s family. **c**, tmRNA family. **d**, RNaseP family. **e**, srp family. **f**, grp1 family. **g**, 23s family. **h**, 16s family. **i**, telomerase family.

Supplementary Table 1. Performance metrics for RNA secondary structure prediction models on different benchmark datasets.

		ArchiveII		bpRNA		bpRNA-new		PDB-RNA		Average	
		F_1	WL	F_1	WL	F_1	WL	F_1	WL	F_1	WL
Classical	RNAfold	0.59	0.70	0.53	0.68	0.67	0.76	0.68	0.69	0.62	0.71
	LinearPartition-V	0.61	0.71	0.53	0.68	0.67	0.75	0.67	0.69	0.62	0.71
	RNAstructure	0.57	0.68	0.52	0.67	0.64	0.74	0.66	0.67	0.60	0.69
Hybrid	LinearPartition-C	0.67	0.76	0.60	0.75	0.71	0.81	0.68	0.68	0.66	0.75
	MXfold2	0.80	0.85	0.54	0.74	0.61	0.76	0.31	0.47	0.57	0.71
Deep learning	REDfold	0.98	0.98	0.69	0.82	0.50	0.73	0.38	0.53	0.64	0.76
	UFold	0.93	0.94	0.63	0.74	0.54	0.69	0.58	0.61	0.67	0.75
	sincFold	0.97	0.98	0.68	0.83	0.44	0.72	0.57	0.62	0.67	0.78
RNA-LLM	ERNIE-RNA	0.95	0.95	0.68	0.76	0.67	0.73	0.36	0.40	0.67	0.71
	RNAErnie	0.76	0.74	0.41	0.59	0.38	0.55	0.18	0.31	0.43	0.55
	RiNALMo	0.95	0.95	0.64	0.76	0.43	0.63	0.37	0.42	0.60	0.69
	one-hot	0.57	0.60	0.35	0.58	0.39	0.58	0.34	0.36	0.41	0.53
	RNA-MSM	0.74	0.71	0.42	0.63	0.39	0.61	0.34	0.33	0.47	0.57
	RNABERT	0.62	0.64	0.33	0.55	0.36	0.55	0.24	0.33	0.39	0.52
	RNA-FM	0.91	0.90	0.52	0.65	0.41	0.58	0.25	0.33	0.52	0.61

Supplementary Table 2. Performance metrics for RNA secondary structure prediction models on different RNA families.

		tRNA		5s		tmRNA		RNaseP		srp		grp1		23s		16s		telomerase		Average	
		F_1	WL	F_1	WL	F_1	WL	F_1	WL	F_1	WL	F_1	WL	F_1	WL	F_1	WL	F_1	WL	F_1	WL
Classical	RNAfold	0.71	0.78	0.69	0.76	0.43	0.60	0.54	0.68	0.67	0.74	0.56	0.68	0.74	0.80	0.53	0.68	0.48	0.62	0.53	0.63
	LinearPartition-V	0.70	0.76	0.73	0.78	0.42	0.58	0.58	0.70	0.66	0.74	0.57	0.68	0.74	0.82	0.58	0.71	0.45	0.60	0.54	0.64
	RNAstructure	0.73	0.79	0.62	0.72	0.40	0.58	0.54	0.68	0.64	0.72	0.52	0.65	0.68	0.78	0.57	0.70	0.46	0.60	0.52	0.62
Hybrid	LinearPartition-C	0.76	0.82	0.78	0.83	0.38	0.60	0.58	0.71	0.68	0.75	0.61	0.73	0.69	0.77	0.66	0.77	0.51	0.65	0.56	0.66
	MXfold2	0.53	0.67	0.58	0.70	0.41	0.59	0.51	0.66	0.61	0.71	0.48	0.62	0.58	0.71	0.51	0.68	0.38	0.57	0.46	0.59
Deep learning	REDfold	0.40	0.65	0.49	0.67	0.26	0.56	0.37	0.61	0.17	0.51	0.31	0.60	0.40	0.64	0.36	0.62	0.09	0.53	0.29	0.54
	UFold	0.49	0.68	0.38	0.63	0.35	0.58	0.43	0.64	0.20	0.51	0.44	0.63	0.39	0.61	0.29	0.61	0.19	0.54	0.31	0.54
	sincFold	0.71	0.79	0.46	0.65	0.34	0.58	0.45	0.63	0.24	0.53	0.35	0.60	0.40	0.63	0.41	0.63	0.12	0.57	0.35	0.56
RNA-LLM	ERNIE-RNA	0.84	0.87	0.84	0.85	0.73	0.76	0.72	0.76	0.67	0.73	0.36	0.56	0.56	0.69	0.57	0.72	0.14	0.43	0.54	0.64
	RNAErnie	0.81	0.84	0.55	0.64	0.26	0.46	0.19	0.48	0.12	0.43	0.17	0.40	0.24	0.52	0.15	0.52	0.07	0.40	0.26	0.47
	RiNALMo	0.87	0.88	0.82	0.84	0.70	0.77	0.73	0.74	0.63	0.70	0.64	0.71	0.59	0.67	0.51	0.66	0.09	0.44	0.56	0.64
	one-hot	0.46	0.60	0.28	0.50	0.17	0.35	0.17	0.39	0.15	0.39	0.15	0.37	0.18	0.43	0.16	0.40	0.13	0.36	0.19	0.38
	RNA-MSM	0.34	0.55	0.26	0.48	0.24	0.45	0.20	0.44	0.12	0.41	0.18	0.43	0.21	0.45	0.14	0.45	0.07	0.41	0.18	0.41
	RNABERT	0.46	0.60	0.22	0.49	0.15	0.41	0.17	0.41	0.12	0.42	0.15	0.41	0.16	0.42	0.15	0.42	0.09	0.39	0.17	0.40
	RNA-FM	0.79	0.83	0.56	0.63	0.28	0.56	0.35	0.52	0.21	0.51	0.13	0.42	0.22	0.56	0.17	0.52	0.13	0.43	0.28	0.50

References

1. J Demšar, Statistical comparisons of classifiers over multiple data sets. *Journal of Machine Learning Research* **7**, 1–30 (2006).

2. A Benavoli, G Corani, F Mangili, Should we really use post-hoc tests based on mean-ranks? *Journal of Machine Learning Research* **17**, 1–10 (2016).
