# Supplementary figures and images for: Comprehensive benchmarking of large language models for RNA secondary structure prediction
Source: Brief Bioinform. 2025 Apr 10;26(2):bbaf137. doi: 10.1093/bib/bbaf137 (PMC11982019; doi:10.1093/bib/bbaf137)

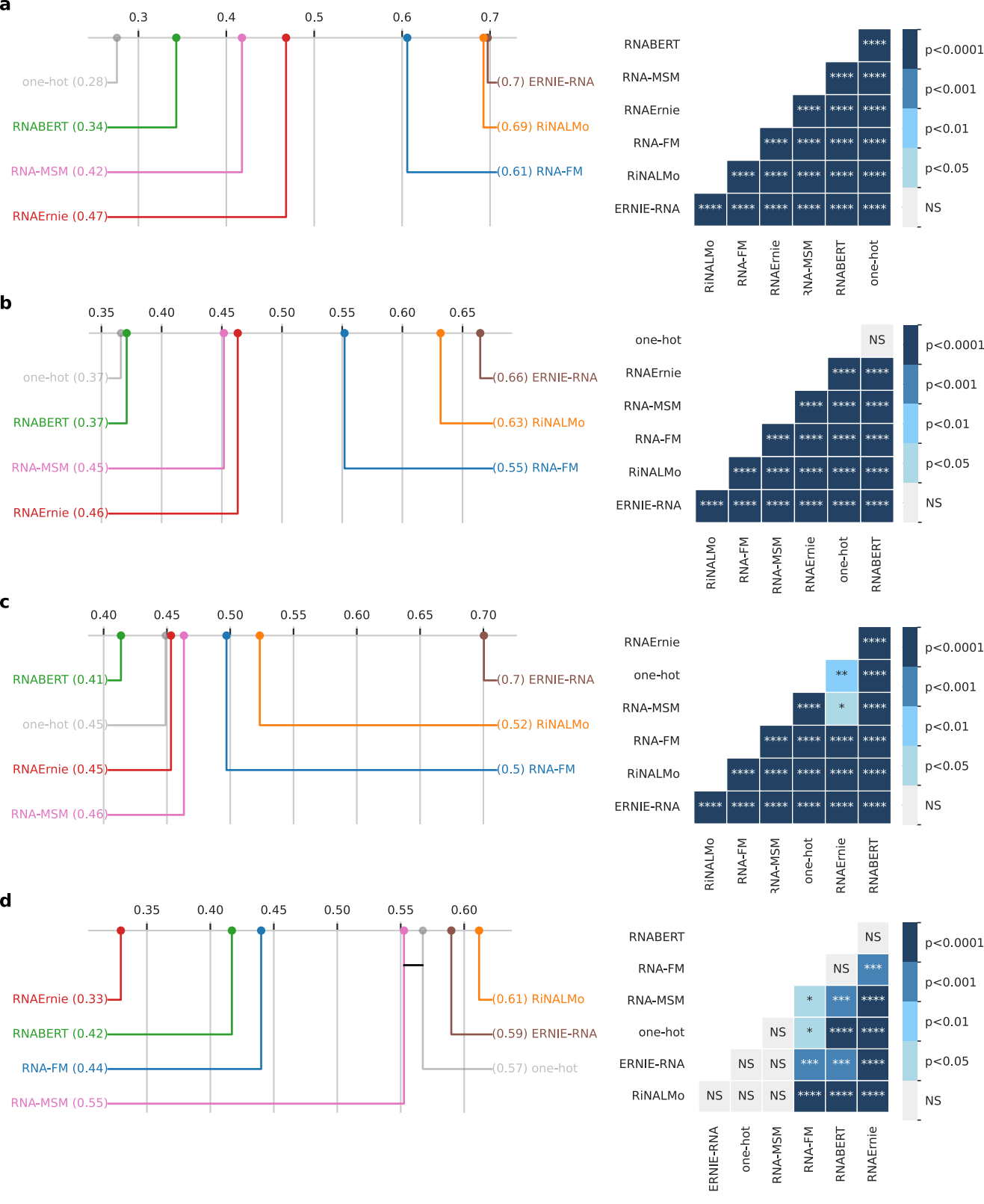

Supplement: fig_S1_v1_bbaf137 [file fig_s1_v1_bbaf137.pdf]

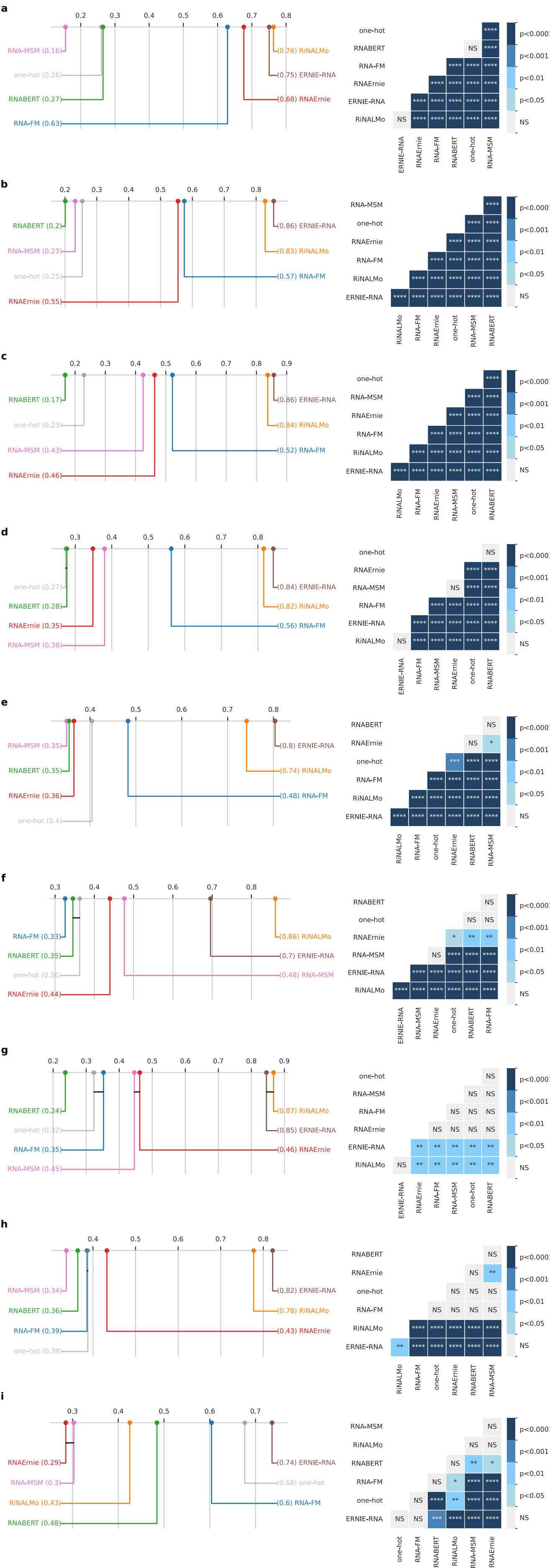

Supplement: fig_S2_v1_bbaf137 [file fig_s2_v1_bbaf137.pdf]
